# Quality of life after risk reducing mastectomy in a Portuguese cohort of *BRCA* pathogenic/likely pathogenic variant carriers

**DOI:** 10.1007/s00432-025-06231-9

**Published:** 2025-05-30

**Authors:** Maria Raposo, Bárbara Peleteiro, André Magalhães, Sandra Torres, Inês Insua-Pereira, Raquel Guimarães, Luzia Garrido, Susy Costa, José Luis Fougo

**Affiliations:** 1 Faculdade de Medicina, Universidade do Porto, Porto, Portugal; 2 Departamento de Ciências da Saúde Pública e Forenses e Educação Médica, Faculdade de Medicina, Universidade do Porto, Porto, Portugal; 3 Breast Center, Unidade Local de Saúde de São João, Porto, Portugal; 4 EPIUnit-Instituto de Saúde Pública, Universidade do Porto, Porto, Portugal; 5 Laboratory for Integrative and Translation Research in Population Health (ITR), Universidade do Porto, Porto, Portugal; 6 Unidade de Saúde Familiar São João, Unidade Local de Saúde Entre Douro e Vouga, São João da Madeira, Portugal; 7 Department of Plastic and Reconstructive Surgery and Burn Unit, Unidade Local de Saúde de São João, Porto, Portugal; 8 Medical Genetic Service and Breast Center, Unidade Local de Saúde de São João, Porto, Portugal; 9 Expression Regulation in Cancer Group, I3S - Institute for Research and Innovation in Health at the University of Porto, Porto, Portugal; 10 Departamento de Cirurgia e Fisiologia, Faculdade de Medicina, Universidade do Porto, Porto, Portugal

**Keywords:** *BRCA* variant carriers, Breast cancer risk, Patient-reported outcome measures, Risk management options, Shared-decision making

## Abstract

**Purpose:**

Women with pathogenic/likely pathogenic (P/LP) variants in *BRCA1/2* genes have an increased lifetime risk of breast and ovarian cancer. Cancer risk management options include intensive breast surveillance (IBS) and risk reducing mastectomy (RRM). This study aims to compare the effect of these strategies on quality of life, anxiety, and depression to enhance shared decision-making.

**Methods:**

We retrospectively analysed clinical records of 221 women with P/LP variants in *BRCA1/2* genes, from 2007 to 2024. A total of 169 questionnaires containing *Hospital Anxiety and Depression Scale (HADS*) and *BREAST-Q* were sent, from May to September 2024. Ninety-nine women, 48 who had undergone RRM and 51 who had opted for IBS, completed the questionnaires. Patient-reported outcome measures (PROMs) were compared based on their choice.

**Results:**

Significant differences were found in age at genetic testing and personal history of breast cancer between the groups. In *BREAST-Q*, the IBS group reported higher scores, with statistically significant differences for *Satisfaction with Breasts* and *Physical Well-Being: Chest.* These differences were only observed in the group of women without personal breast cancer history who underwent RRM.

**Conclusions:**

No significant differences were found in psychologic distress levels between the IBS and RRM group. Although RRM is an effective method for reducing breast cancer risk in women with P/LP variants in *BRCA1/2* genes, carriers should be informed of its impact on quality of life. Notably, once a woman is diagnosed with breast cancer, these differences lose effect.

## Introduction

Hereditary breast cancers syndromes are estimated to underlie 5% to 10% of all breast cancers. Pathogenic germline variants in tumour suppressor genes, specifically *BRCA1* and *BRCA2*, account for 25 to 30% of hereditary cases (Baretta et al. 2016). Pathogenic/likely pathogenic (P/LP) variants in *BRCA1*/2 genes have an autosomal dominant transmission pattern. *BRCA* genes are tumour suppressor genes, whose mutation leads to impaired DNA repair during homologous recombination (Narod and Foulkes 2004). Such variants are associated with a risk of breast cancer to age 80 of 72% for *BRCA1* variant carriers and 69% for *BRCA2* variant carriers, while the general population has a lifetime risk of 12% (Metcalfe et al. 2019). Carriers have a higher risk of bilateral and multicentric breast cancer as well as a higher risk of developing cancer in other organs, such as ovarian cancer (Kuchenbaecker et al. 2017).

Since the risk of developing breast cancer increases with age, there is particular concern regarding timing and selection of risk management strategies in this population. Strategies include risk reducing mastectomy (RRM) or intensive breast surveillance (IBS). Management recommendations for hereditary breast cancer syndromes differ across Europe. For carriers of P/LP variants in *BRCA1/2* genes, all guidelines recommend incorporating breast magnetic resonance imaging (MRI) into IBS, typically starting between ages 25–30, with mammography added at 30–40 years. Clinical breast examination is advised from as early as age 18. While no guideline issues a categorical recommendation for RRM, it is consistently presented as an effective risk-reducing option to be considered within an informed decision-making framework (Marmolejo et al. 2021).

RRM decreases the risk of developing breast cancer in carriers of P/LP variants in *BRCA1/2* genes by more than 90% (Franceschini et al. 2019). RRM may be associated with a decrease in psychological distress, although sometimes accompanied by body image, psychosocial, and psychosexual problems, as well as possible surgical morbidity (den Heijer et al. 2012).

Although a survival benefit has been demonstrated for carriers of P/LP variants in *BRCA1* undergoing either IBS or RRM, with RRM being associated with significantly lower breast cancer-specific mortality (Heemskerk-Gerritsen et al. 2019; Lubinski et al. 2024), the choice between IBS or RRM is based on the carrier’s preferences and beliefs. To assist the carrier in their decision-making process it is important to explain the pros and cons of both options. Carriers who are considering RRM should be informed not only about the impact of surgery on survival and breast cancer risk, but on expected quality of life outcomes as well (van Egdom et al. 2020).

According to the principles of value-based healthcare, these can be provider-reported or patient-reported outcomes (PROs). PROs, which are obtained directly from patients using validated questionnaires (patient-reported outcome measures or PROMs), provide insight into patients' quality of life and functional status. PROs are important for understanding long-term effect on quality of life and can serve as a resource for carriers when making decisions about breast cancer risk management (Di Maio et al. 2022). Despite their importance, PROs following either RRM or ongoing IBS among women carriers of P/LP variants in *BRCA1/2* genes vary among studies (Luque Suárez et al. 2022).

The aim of this study was to compare the effect of RRM and IBS on quality of life, anxiety, and depression in women carriers of P/LP variants in *BRCA1/2* genes who are followed in a High-Risk Breast Clinic in a Portuguese Breast Centre.

## Materials and methods

Women carriers of P/LP variants in *BRCA1/2* genes were retrospectively identified at a High-Risk Breast Clinic at the Breast Centre of the *Unidade Local de Saúde de São João*. The cohort consisted of 221 women, diagnosed with P/LP variants in *BRCA1/2* genes in the Genetic Department of the *Faculdade de Medicina da Universidade do Porto* and observed at the High-Risk Breast Clinic of a public university hospital's Breast Centre from November 30th, 2007, to September 27th, 2024. In this High-Risk Breast Clinic, referral and patient management are based on NCCN and ESMO guidelines: individuals meeting NCCN testing criteria are referred, and those eligible are offered RRM or IBS. IBS consists of a clinical breast examination every six months, with an annual mammogram alternating every six months with breast MRI, starting 5 years before the youngest affected family member or, at the latest, between ages 25–30 (Sessa et al. 2023; NCCN guidelines for detection, prevention, and risk reduction. Available from: https://www.nccn.org/professionals/physician_gls/#detection). Inclusion criteria required participants to be aged 18 years or older and to have opted for either RRM or IBS.

Clinical information was obtained through the patient’s digital records: age at first appointment at the High-Risk Breast Clinic, *BRCA1/2* variant, marital status, education level, smoking status, body mass index (BMI), age at genetic testing, personal and family history of breast cancer, personal and family history of ovarian cancer, personal history of other types of cancer and risk management option.

Women with personal history of breast cancer were considered affected. In this group, characteristics concerning breast cancer were collected: age at diagnosis, type of surgery and recurrence. RRM was defined as bilateral risk reducing mastectomy (BRRM) for the unaffected carrier group and as contralateral risk reducing mastectomy (CRRM) for the affected carrier group. Data regarding the timing of breast reconstruction was collected for the affected group and for the RRM group, as immediate breast reconstruction (IBR) or delayed breast reconstruction (DBR).

The patients identified were recruited by telephone from March to May 2024. Informed consent was obtained from all individual participants included in the study*.* Patients were asked to provide an email address through which two PROM digital questionnaires were to be sent: the *Hospital Anxiety and Depression Scale* (*HADS*) (Zigmond and Snaith 1983) and the *BREAST-Q version 2.0* reconstruction module (preoperative section for the IBS and RRM group and postoperative for the RRM group) (Pusic et al. 2009). Both PROMs were web-based questionnaires and administered through the software program “*Google Forms*”, in their validated Portuguese versions (Pais-Ribeiro et al. 2007; Pinto et al. 2023). Patients unable to answer the questionnaire digitally were asked if a letter containing an informed consent form and the two questionnaires could be sent to their home address. If the questionnaires remained uncompleted, a monthly reminder was sent through email. If participants had not responded in 2 months, they were contacted by telephone and asked to complete the questionnaires. Data regarding the results of the questionnaires was collected from May to September 2024. PROM scores were calculated according to the questionnaires’ scoring manuals (Zigmond and Snaith 1983; *BREAST-Q Version 2.0*© User’s Guide. Available from: https://qportfolio.org/wp-content/uploads/2018/12/BREAST-Q-USERS-GUIDE.pdf).

*BREAST-Q version 2.0* scores are computed by adding response items together and directly converting the sum to a score, usually from 0–100. There are questions considered as “stand-alone”, where the patient’s response is taken as the score for each item. These include *Satisfaction with Abdomen* (maximum score of 4) in the preoperative section; in the postoperative section: *Physical Well-being: Chest* (maximum score of 3), *Satisfaction with Implants* (maximum score of 8), *Satisfaction with Nipple Reconstruction* (maximum score of 4), *Adverse Effects of Radiation* (ranging from 6 to 36) and *Satisfaction with Abdomen* (maximum score of 16). Higher scores reflect a better satisfaction or QoL, except for *Adverse Effects of Radiation*, where lower scores reflect a better outcome.

*HADS* consists of two sections: one assessing anxiety (*HADS-A*) and another assessing depression (*HADS-D*). Each section has a scoring range of 0 to 21. The defined cut-offs for both anxiety and depression are as follows: Normal (< 8), Moderate (8–12), and Severe (> 12). Psychological evaluation is recommended for scores between 8 and 12, while psychiatric evaluation is indicated for scores above 12 (Hinz and Brähler 2011).

Data collected from clinical records was analysed using SPSS version 26.0 (IBM Corp., Armonk, NY, USA). Descriptive statistics were used to summarize the main characteristics of the sample. Quantitative variables were summarized by measures of central tendency (mean or median, as applicable) and dispersion (standard deviation) and qualitative variables were summarized by absolute (*N*) and relative (%) frequency. The Chi-square or Fisher’s exact test, as appropriate, was used for comparison of proportions, while Student’s *t* test or Mann–Whitney test was used for comparison of means or medians, respectively. A *p* value < 0.05 was considered statistically significant. The PROM scores of participants were compared to evaluate differences according to risk management option chosen, either RRM or IBS.

## Results

**Fig. 1:**
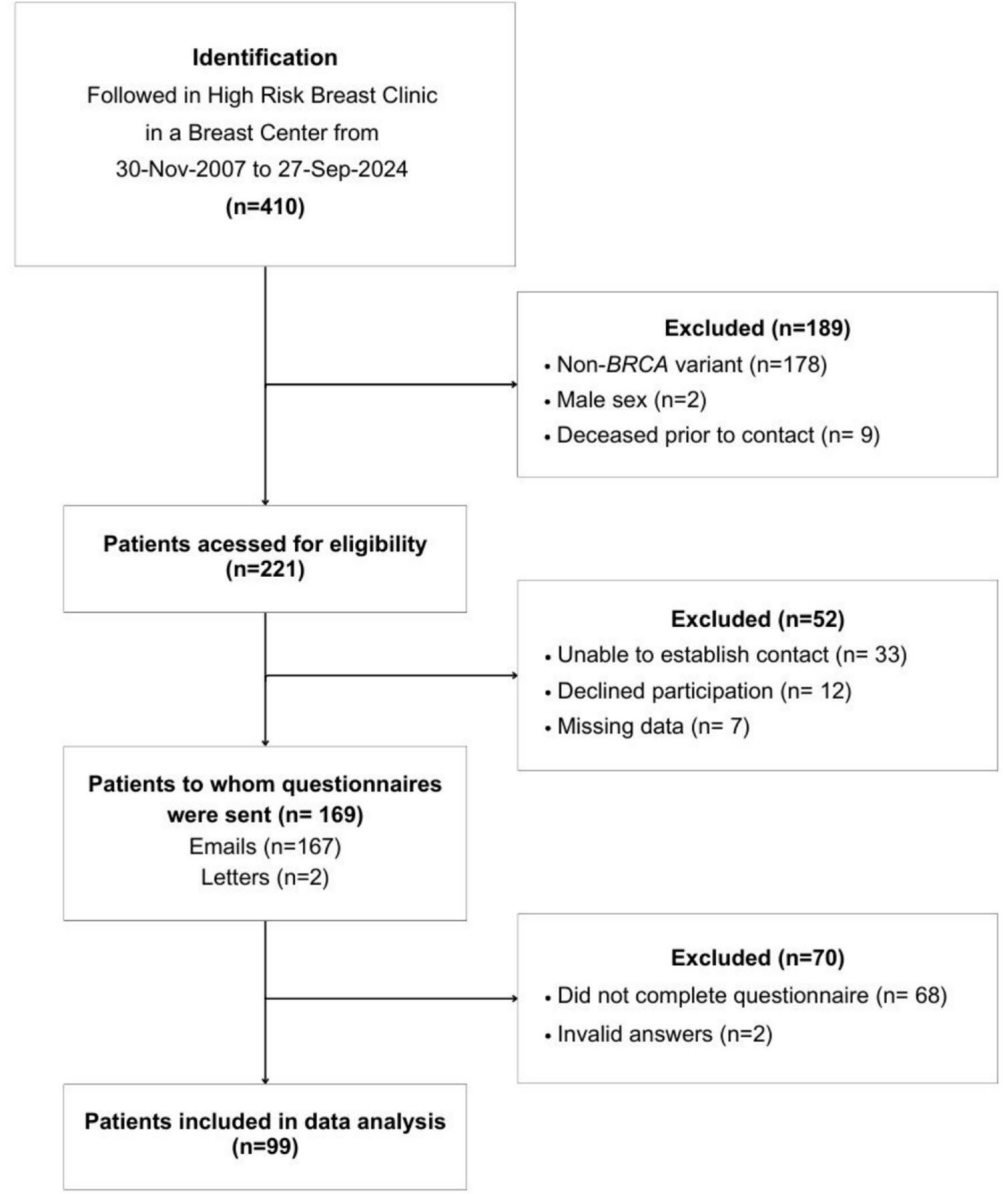
Flowchart of participant selection

A total of 410 patients followed at a High-Risk Breast Clinic were identified (Fig. 1). One hundred and eighty-nine (46.1%) patients were excluded due to non-P/LP *BRCA 1/2* variants (n = 178, 43.4%), male sex (n = 2, 0.5%) and being deceased prior to contact (n = 9, 2.2%).

Between March 2024 and May 2024, 221 eligible women were contacted via telephone. Among the 52 (19.2%) women excluded from the study, 12 (5.4%) had declined participation. Of 169 questionnaires sent, 58.6% were completed. Sixty-eight (40.2%) women did not reply despite verbal consent being obtained through telephone and two (1.2%) responders gave invalid answers.

Overall, 99 carriers of *BRCA1/2* P/LP variants, who had been followed in the High-Risk Clinic for a median period of 48 months (0–181 months) from November 2007 to September 2024, completed the questionnaire.

### Patient characteristics

Table 1 summarizes the characteristics of the patients in the sample. The mean age at the time of genetic testing was 42.7 years (range: 18–75). Of the 99 women, 42 (42.8%) were carriers of *BRCA1* P/LP variants and 57 (57.6%) were carriers of *BRCA2* P/LP variants.

**Table 1:** Patient characteristics according to risk management option chosen

	Total [n = 99]	Risk reducing mastectomy Group [n = 48] N (%)	Intensive breast surveillance Group [n = 51] N (%)	*p* value
Age at genetic testing, years [mean (range)]^a^	42.7 (18.0–75.0)	39.5 (20.0–59.0)	45.5 (18.0–75.0)	0.018
Altered gene				
*BRCA1*	42 (42.4)	22 (45.8)	20 (39.2)	0.506
*BRCA2*	57 (57.6)	26 (54.2)	31 (60.8)	
Parity^b^				
0	28 (28.9)	16 (33.3)	12 (24.5)	0.337
≥ 1	69 (71.1)	32 (66.7)	37 (75.5)	
Marital status^c^				
Married/union in fact	51 (62.2)	25 (61.0)	26 (63.4)	0.820
Widowed/divorced/single	31 (37.8)	16 (39.0)	15 (36.6)	
Education level^d^				
Elementary school	9 (16.1)	6 (19.4)	3 (12.0)	0.867
High school	15 (26.8)	8 (25.8)	7 (28.0)	
University	32 (57.1)	17 (54.8)	15 (60.0)	
Smoking status^e^				
Non/ex-smoker	60 (82.2)	30 (81.1)	30 (83.3)	0.801
Current smoker	13 (17.8)	7 (18.9)	6 (16.7)	
Body mass index^f^				
Underweight/normal weight	22 (44.0)	10 (43.5)	12 (44.4)	0.945
Overweight/obesity	28 (56.0)	13 (56.5)	15 (55.6)	
Personal history of breast cancer				
No	55 (55.6)	19 (39.6)	36 (70.6)	0.002
Yes	44 (44.4)	29 (60.4)	15 (29.4)	
Age at diagnosis, years [mean (range)]	40.2 (24.0–70.0)	38.2 (24.0–70.0)	44.7 (28.0–64.0)	0.041
Family history of breast cancer				
No	19 (19.2)	11 (22.9)	8 (15.7)	0.361
Yes	80 (80.8)	37 (77.1)	43 (84.3)	
Personal history of ovarian cancer^g^				
No	94 (95.9)	44 (93.6)	50 (98.0)	0.347
Yes	4 (4.1)	3 (6.4)	1 (2.0)	
Age at diagnosis, years [mean (range)]	36.3 (23.0–42.0)	40.7 (39.0–42.0)	23 (-)	-
Family history of ovarian cancer^h^				
No	87 (88.8)	43 (91.5)	44 (86.3)	0.414
Yes	11 (11.2)	4 (8.5)	7 (13.7)	
Bilateral salpingoophorectomy				
No	46 (46.5)	22 (45.8)	24 (47.1)	0.903
Yes	53 (53.5)	26 (54.2)	27 (52.9)	
Personal history of other type of cancer^i^				
No	91 (93.8)	42 (91.3)	49 (96.1)	0.418
Yes	6 (6.2)	4 (8.7)	2 (3.9)	
Age at breast cancer risk management choice [median (range)]	42.0 (19.0–75.0)	40.5 (27.0–70.0)	44.0 (19.0–75.0)	0.201

^a^Unknown for 1 patient in the IBS group; unknown for 5 patients in the RRM group and 4 patients in the IBS group

^b^Unknown for 2 patients in the IBS group

^c^Unknown for 7 patients in the RRM group and 10 patients in the IBS group

^d^Unknown for 17 patients in the RRM group and 26 patients in the IBS group

^e^Unknown for 11 patients in the RRM group and 15 patients in the IBS group

^f^Unknown for 25 patients in the RRM group and 24 patients in the IBS group

^g^Unknown for 1 patient in the RRM group

^h^Unknown for 1 patient in the RRM group

^i^Unknown for 2 patients in the RRM group

Of the 99 women with P/LP variants in *BRCA1/2*, 44 (44.4%) had personal history of breast cancer with the mean age at diagnosis being 40.2 years (range: 24–70), and 4 (4.1%) had personal history of ovarian cancer with the mean age at diagnosis being 36.3 years (range: 23–42). Bilateral salpingoophorectomy had been performed by 53 (53.5%) participants, of which 52 (98.1%) were for risk reduction purpose. Regarding family history, 80 (80.8%) women had family history of breast cancer, and 11 (11.2%) women had family history of ovarian cancer.

After genetic detection of a *BRCA1/2* P/LP variant, carriers were faced with the decision to opt for a risk management option, with 48 (48.5%) women selecting RRM and 51 (51.5%) women selecting IBS. Participants in the RRM group were younger than those in the IBS group when choosing a risk management strategy, although this difference was not statistically significant (40.5 vs 44.0 years, *p* = 0.201). The RRM group consisted of 19 (39.6%) women who underwent BRRM and 29 (60.4%) women with previous personal breast cancer history who underwent CRRM. Of the 48 women who underwent RRM, 45 (93.8%) had breast reconstruction, and of these, three did not opt for IBR. Of the 51 women who opted for IBS, 1 (2.0%) woman in the unaffected group developed bilateral breast cancer, and in the group with previous personal breast cancer history, 6 (11.2%) women developed breast cancer in the contralateral breast during surveillance. No participants in the RRM group developed breast cancer during follow-up.

### Patient characteristics according to Breast Cancer Risk Management Option Chosen

Table 1 also compares patient characteristics between the RRM and IBS groups. The participants in the RRM group were younger than those in the IBS group at genetic testing (39.5 vs 45.5 years, *p* = 0.018).

There was a higher proportion of women with personal history of breast cancer who opted for RRM in comparison to those without personal history of breast cancer (60.4% vs 39.6%, *p* = 0.002). Among the women with personal history of breast cancer, those in the RRM group were younger at diagnosis than those in the IBS group (38.2 vs 44.7 years, *p* = 0.041).

### Patient scores according to Breast Cancer Risk Management Option Chosen

Table 2 compares patient scores on *BREAST-Q* between the RRM and IBS groups. The RRM group scored lower than the IBS group in most scales of *BREAST-Q*, although these differences were not statistically significant. Only on the *Psychosocial Well-Being* scale the participants in the RRM group scored higher than those in the IBS group, even though this difference was not statistically significant (67.5 vs 64.0 out of 100, *p* = 0.793). On the contrary, women in the IBS group scored higher in both the *Satisfaction with Breasts* scale (58.0 vs 71.0 out of 100, *p* = 0.012) and *Physical Well-Being: Chest* scale (72.0 vs 85.0 out of 100, *p* = 0.017), and these differences were statistically significant. Although not statistically significant, *Sexual Well-Being* scores were also higher in the IBS group (46.0 vs 59.0 out of 100, *p* = 0.263).

**Table 2:** Patient scores on *BREAST-Q version 2.0* according to risk management option chosen

Score [median (interquartile range)]	Total [n = 99]	Maximum Score	Risk reducing mastectomy group [n = 48] N (%)	Intensive breast surveillance group [n = 51] N (%)	*p* value
Preoperative					
Satisfaction with breasts	64.0 (47.0)	100.0	58.0 (34.0)	71.0 (42.0)	0.012
Satisfaction with abdomen	3.0 (N/A**)	4.0	3.0 (1.0)	3.0 (N/A**)	0.122
Physical well-being: abdomen	69.0 (48.0)	100.0	66.0 (53.0)	76.0 (41.0)	0.128
Pre and postoperative					
Psychosocial well-being	66.0 (31.0)	100.0	67.5 (32.0)	64.0 (31.0)	0.793
Sexual well-being	53.0 (30.0)	100.0	46.0 (32.0)	59.0 (27.0)	0.263
Physical well-being: chest	80.0 (40.0)	100.0	72.0 (41.0)	85.0 (32.0)	0.017
Physical well-being: back and shoulder	62.0 (35.0)	100.0	60.0 (28.8)	69.0 (37.0)	0.215

Score [median (interquartile range)]	Respondents N (%)	Maximum score	Risk reducing mastectomy group [n = 48] N (%)
Postoperative			
Satisfaction with breasts	45 (93.8)	100.0	64.0 (38.0)
Satisfaction with implants	36 (75.0)	8.0	6.0 (3.0)
Satisfaction with nipple reconstruction	10 (20.8)	4.0	2.5 (4.0)
Satisfaction with back	7 (14.6)	100.0	47.0 (32.0)
Physical well-being: abdomen	5 (10.4)	100.0	100.0 (36.0)
Physical well-being: chest	44 (97.8)	3.0	3.0 (N/A**)
Satisfaction with abdomen	5 (10.4)	12.0	9.0 (1.0)
Adverse effects of radiation	2 (4.2)	18.0	9.0 (N/A*)
Patient experience			
Satisfaction with information	43 (89.6)	100.0	56.0 (39.0)
Satisfaction with surgeon	44 (97.8)	100.0	100.0 (14.0)
Satisfaction with medical team	44 (97.8)	100.0	100.0 (6.8)
Satisfaction with office staff	44 (97.8)	100.0	100.0 (5.3)

N/A—not applicable

N/A*—IQR not available as only the 50th percentile is possible to calculate

N/A**—all participants provided the same response

Since only the participants in the RRM group completed the postoperative section of *BREAST-Q*, only their scores are available. There was a limited number of participants who underwent nipple reconstruction (n = 10), reconstruction with *latissimus dorsi* muscle flap (n = 7) or reconstruction with transverse rectus abdominus myocutaneous (TRAM)/ deep inferior epigastric artery perforator flap (DIEP) (n = 5). Only 36 women underwent reconstruction with implants. In the *Patient Experience* section, participants reported high satisfaction with the surgeon, medical team and office staff (100.0 out of 100.0 for the three scales) but low satisfaction with the information received about the surgery (56.0 out of 100.0).

Table 3 compares *HADS* scores between the RRM and IBS groups. The median score was lower in the RRM group than in the IBS group in both the *HADS-A* scale (7.0 vs 8.0, *p* = 0.598) and in the *HADS-D* scale (4.0 vs 5.0, *p* = 0.201). Therefore, a higher proportion of participants in the IBS group was classified as Moderate or Severe in the *HADS-A* and *HADS-D* scales. Nevertheless, these differences across the groups were not statistically significant.

**Table 3:** Patient scores on *HADS* according to risk management option chosen

Score	Total [n = 99] N (%)	Risk reducing mastectomy group [n = 48] N (%)	Intensive breast surveillance group [n = 51] N (%)	*p* value
*HADS-A* [median (IQR)]	7.0 (7.0)	7.0 (7.0)	8.0 (7.0)	0.598
Normal	54 (54.5)	29 (60.4)	25 (49.0)	0.404
Moderate	33 (33.3)	15 (31.3)	18 (35.3)	
Severe	12 (12.1)	4 (8.3)	8 (15.7)	
*HADS-D* [median (IQR)]	5.0 (5.0)	4.0 (5.0)	5.0 (6.0)	0.201
Normal	75 (75.8)	39 (81.3)	36 (70.6)	0.450
Moderate	21 (21.2)	8 (16.7)	13 (25.5)	
Severe	3 (3.0)	1 (2.1)	2 (3.9)	

### Patient scores according to previous personal history of breast cancer

Table 4 compares patient scores on *BREAST-Q* according to risk management strategy chosen, stratified by personal history of breast cancer. Scores of the participants with personal history of breast cancer showed no statistically significant differences according to strategy chosen. On the contrary, participants with no personal history of breast cancer who selected IBS scored higher on the *Satisfaction with Breasts* scale (58.0 vs 71.0 out of 100, *p* = 0.031) and the *Physical Well-being: Chest* scale (72.0 vs 92.0 out of 100, *p* = 0.006) than those who selected RRM.

**Table 4:** Patient scores on *BREAST-Q version 2.0* according to Personal History of Breast Cancer when choosing risk management option

Score [median (range)]	Maximum score	No personal history of breast cancer [n = 55] N (%)		*p* value	Personal history of breast cancer [n = 44] N (%)		*p* value
		Risk reducing mastectomy group [n = 19]	Intensive breast surveillance group [n = 36]		Risk reducing mastectomy group [n = 29]	Intensive breast surveillance group [n = 15]	
Preoperative							
Satisfaction with breasts	100.0	58.0 (43.0)	71.0 (42.0)	0.031	58.0 (32.0)	58.0 (42.0)	0.421
Satisfaction with abdomen	4.0	3.0 (N/A**)	3.0 (N/A**)	0.574	3.0 (3.0)	3.0 (N/A**)	0.383
Physical well-being: abdomen	100.0	69.0 (51.0)	76.0 (41.0)	0.474	59.0 (42.0)	69.0 (48.0)	0.445
Pre and postoperative							
Psychosocial well-being	100.0	69.0 (30.0)	64.0 (30.0)	0.859	66.0 (32.0)	74.0 (48.0)	0.371
Sexual well-being	100.0	46.0 (36.0)	59.0 (25.0)	0.404	48.0 (25.0)	53.0 (50.0)	0.833
Physical well-being: chest	100.0	72.0 (42.0)	92.0 (19.0)	0.006	72.0 (37.0)	68.0 (31.0)	0.280
Physical well-being: back and shoulder	100.0	71.0 (41.0)	72.0 (30.0)	0.455	58.0 (26.5)	53.0 (32.0)	0.327

Score [median (range)]	Maximum score	No personal history of breast cancer [n = 55]		Personal history of breast cancer [n = 44]	
		Risk reducing mastectomy [n = 19]	Respondents N (%)	Risk reducing mastectomy [n = 29]	Respondents N (%)
Postoperative					
Satisfaction with breasts	100.0	54.5 (46.0)	18 (94.7)	65.0 (34.0)	27 (93.1)
Satisfaction with implants	8.0	6.0 (2.0)	14 (73.7)	6.0 (4.0)	22 (75.9)
Satisfaction with nipple reconstruction	4.0	1.5 (N/A*)	2 (10.5)	3.0 (4.0)	8 (27.6)
Satisfaction with back	100.0	50.0 (N/A*)	2 (10.5)	47.0 (49.0)	5 (17.2)
Physical well-being: abdomen	100.0	100.0 (27.0)	4 (21.2)	64.0	1 (3.5)
Physical well-being: chest	3.0	3.0 (N/A**)	18 (94.7)	3.0 (1.0)	26 (89.7)
Satisfaction with abdomen	12.0	9.0 (2.0)	4 (21.2)	64.0	1 (3.5)
Adverse effects of radiation	18.0	N/A	N/A	9.0 (N/A*)	2 (6.9)
Patient experience					
Satisfaction with information	100.0	57.0 (51.3)	18 (94.7)	56.0 (34.0)	25 (86.2)
Satisfaction with surgeon	100.0	100.0 (14.0)	18 (94.7)	100.0 (22.8)	26 (89.7)
Satisfaction with medical team	100.0	100.0 (2.3)	18 (94.7)	100.0 (10.5)	26 (89.7)
Satisfaction with office staff	100.0	100.0 (N/A**)	18 (94.7)	100.0 (8.5)	26 (89.7)

N/A—not applicable

N/A*—IQR not available as only the 50th percentile is possible to calculate

N/A**—all participants provided the same response

Table 5 compares patient scores on *HADS* according to risk management strategy chosen, stratified by personal history of breast cancer. Scores on both scales were higher among participants with personal history of breast cancer and a higher proportion of participants in the IBS group was classified as Moderate or Severe on both scales. Nevertheless, these differences across groups were not statistically significant.

**Table 5:** Patient scores on *HADS* according to personal history of breast cancer when choosing risk management option

Score	No personal history of breast cancer [n = 55] N (%)		*p* value	Personal history of breast cancer [n = 44] N (%)		*p* value
	Risk reducing mastectomy [n = 19]	Intensive breast surveillance [n = 36]		Risk reducing mastectomy [n = 29]	Intensive breast surveillance [n = 15]	
*HADS-A* [median (IQR)]	5.0 (6.0)	7.0 (7.0)	0.394	7.0 (6.0)	8.0 (3.0)	0.551
Normal	12 (63.2)	20 (55.6)	0.300	17 (58.6)	5 (33.3)	0.242
Moderate	7 (36.8)	11 (30.6)		8 (27.6)	7 (46.7)	
Severe	0 (0.0)	5 (13.9)		4 (13.8)	3 (20.0)	
*HADS-D* [median (IQR)]	2.0 (5.0)	5.0 (7.0)	0.182	4.0 (4.0)	5.0 (4.0)	0.382
Normal	16 (84.2)	26 (72.2)	0.755	23 (79.3)	10 (66.7)	0.468
Moderate	2 (10.5)	8 (22.2)		6 (20.7)	5 (33.3)	
Severe	1 (5.3)	2 (5.6)		0 (0.0)	0 (0.0)	

## Discussion

Carriers of P/LP variants in *BRCA1/2* genes face a complex decision regarding breast cancer risk management. PROs provide insight on the effect of available cancer risk management strategies on quality of life and can improve shared decision-making. This study aimed to compare PROs in women carriers of P/LP variants in *BRCA1/2* according to their choice of breast cancer risk management.

A growing body of literature has compared PROs in carriers of P/LP variants in *BRCA1/2* genes opting between IBS or RRM. However, findings remain difficult to interpret due to heterogeneity in study designs, particularly due to the use of varying PROM instruments, including non-standardized questionnaires (Razdan et al. 2016).

In terms of body image, van Egdom et al. (2020) reported that unaffected carriers who underwent RRM tended to have lower *BREAST-Q* scores in the *Physical Well-Being: Chest* scale but scored higher in the *Psychosocial Well-Being* scale when compared to unaffected carriers who underwent IBS. Another study noted significant differences between affected and unaffected carriers across *BREAST-Q* domains, particularly in psychosocial and sexual well-being (Herold et al. 2022). *BREAST-Q* scores have been reported to vary over time after RRM and reconstruction, but results differ across studies (Brandberg et al. 2008; Razdan et al. 2016; Luque Suárez et al. 2022; Myers et al. 2023).

Regarding psychological outcomes, van Egdom et al. (2020) found no significant differences in anxiety or depression levels between IBS and RRM groups among unaffected carriers, though this was a cross-sectional analysis. In contrast, a longitudinal German study assessed anxiety and depression levels at three time points over an eight-month period following the disclosure of genetic test results. Baseline anxiety levels were higher in women choosing RRM but decreased over time, whereas anxiety increased among those choosing IBS. Depression scores remained stable across both groups (Dick et al. 2022). These findings are consistent with previous research conducted in women at high risk for hereditary breast cancer, where anxiety tends decrease over time after RRM, while depression remains relatively stable (Brandberg et al. 2008; McCarthy et al. 2017). Despite the limitations of our study, our findings seem to be consistent with previous research, showing similar trends in body image and psychological outcomes.

Participants in the RRM group were younger than those in the IBS group at genetic testing. This is in line with guidelines and findings reported in previous studies (van Egdom et al. 2020; Sessa et al. 2023; Torres et al. 2023). The benefits of RRM are most significant when performed after the age of 30, as the cumulative risk of breast cancer before this age is approximately 4%. However, RRM is generally recommended before the age of 55, as evidence supporting its benefit beyond this age remains weak (Sessa et al. 2023). Our group typically advises RRM once women complete their reproductive project or a few years before the youngest case of breast cancer diagnosis in the carrier’s family. Of course, patient preference is the determinant factor. In our study population, women with personal history of breast cancer were more likely to opt for RRM over IBS. In fact, previous breast cancer history outstands all other variables regarding the choice of RRM over IBS. This may reflect an increased perception of the reality of the disease and motivation to undergo risk reducing surgery. Younger age at diagnosis also influenced this decision. Surprisingly, family history of breast cancer did not appear to influence this decision.

In our study, the definition of RRM included BRRM in unaffected women and CRRM in affected women. Of the 48 women included in the RRM group, 19 (39.6%) underwent BRRM and 29 (60.4%) underwent CRRM, which aligns with personal history of breast cancer in the RRM group. This definition of RRM has been widely accepted in recent research (Carbine et al. 2018; Sessa et al. 2023), although not always clear in the literature.

No statistically significant differences were observed in the subgroup analysis between *BRCA1/2* P/LP variant carriers except for family history of ovarian cancer (data not shown). In a previous retrospective study conducted by our group, which analysed a total of 187 *BRCA1/2* P/LP variant carriers, patient characteristics were described (Torres et al. 2023). Our current study, based on a smaller subset of this population, reveals characteristics comparable to those of the larger cohort, suggesting that our subset is representative of the population followed in a high-risk consultation.

Although multiple PROM instruments are available, *HADS* was selected since it is a short questionnaire and the most extensively validated scale for screening emotional distress in cancer patients (Vodermaier and Millman 2011; Rojas et al. 2019), while *BREAST-Q* was selected because it is a validated breast-specific instrument that is used worldwide (Pais-Ribeiro et al. 2007; van Egdom et al. 2020; García-Solbas et al. 2021).

Currently, no consensus exists regarding the interpretation of *BREAST-Q* scores. However, a difference of 5 points is regarded as indicative of a small clinical difference, 10 points as a moderate clinical difference, and 20 points as a highly clinically significant difference (Cano et al. 2014). *BREAST-Q* scores are within current normative data for women who have undergone breast surgery (Mundy et al. 2017) and align with previous research, although there are cultural differences between Portuguese and American women, for which these scores were validated. Overall, the IBS group scored higher on *BREAST-Q* than the RRM group, and this difference has been reported in previous studies (van Egdom et al. 2020). We believe women dissatisfied with their breasts and sexual life more easily opt for mastectomy as they may feel they have less to lose from the procedure. These differences appear to be associated with personal history of breast cancer, as they are consistently observed across both scales but exclusively in women with no personal history of breast cancer. While previous studies reported *BREAST-Q* scores in unaffected carriers that are comparable to those of our cohort (van Egdom et al. 2020), our findings are the first to document the absence of such differences in women with a prior breast cancer diagnosis.

In the *Patient Experience* section, participants reported high satisfaction with the surgeon, medical team and office staff but low satisfaction with the information received about the surgery, and this is observed in both groups, irrespective of personal history of breast cancer. These findings suggest a need for greater focus on shared decision-making and improvements in the quality and comprehensiveness of informed consent discussions.

No significant differences were observed in *HADS* scores across groups, and this is in line with other studies’ findings (van Egdom et al. 2020; Luque Suárez et al. 2022), although our median *HADS* scores were higher. A greater proportion of participants in the IBS group scored Moderate or Severe on both scales when compared to those in the RRM group. These differences, however, are not statistically significant, highlighting that women choosing IBS do not seem to be more anxious or depressed due to their medical follow-up or higher risk of developing cancer. Meneses (2020) conducted a study in our High-Risk Breast Clinic, and although it only included affected carriers of P/LP variants in the *BRCA1/2* genes, results were comparable. We hypothesized that the presence of a multidisciplinary team working in the same location may have served as a protective factor in these affected carriers. For the unaffected group, however, we hypothesize that these results may be explained by a limited perception of the actual risk of developing breast cancer among carriers of P/LP variants in *BRCA1/2* genes, potentially due to the absence of effective consent discussions and risk communication with healthcare providers.

Due to the retrospective nature of the study and the absence of baseline PROM scores, it is challenging to determine whether women with higher levels of anxiety and/or depression may be less likely to undergo RRM, or if the better psychological outcomes found in those who opted for RRM are a consequence of the surgery itself. As anticipated, women with a personal history of breast cancer tend to score higher on both the anxiety and depression scales (Smith 2015). Consequently, a higher proportion of women with personal history of breast cancer was classified as having moderate or severe anxiety and depression compared to those without personal breast cancer history. This is an expected finding and further reinforces the clinical validity of our results.

The key question is whether the small reduction in depression and anxiety potentially associated with RRM justifies the possible small decline in quality of life, body image satisfaction, and sexual well-being for the individual carrier of a P/LP variant in *BRCA1/2*. This should be incorporated into the consent discussion, ensuring that the carrier understands both the psychological benefits and the potential drawbacks of the surgery. While RRM is an established and effective method for reducing breast cancer risk, it is crucial that carriers understand the risks of their variant, particularly that of breast cancer, to fully appreciate the benefits of undergoing surgery. This is clearly demonstrated by the substantial impact a prior breast cancer diagnosis has on the carrier’s choice of RRM over IBS. Again, this highlights the need for emphasis on shared decision-making and of informed consent processes.

Our findings should be interpreted in light of several limitations. The main limitations are the small sample size (n = 99) and the heterogeneity regarding cancer diagnosis, both of which may restrict the generalizability of the results. Although previous studies had similar or smaller cohorts (van Egdom et al. 2020; García-Solbas et al. 2021), this remains a constraint. A further limitation is the high number of missing values across key variables, particularly sociodemographic data, which may have limited our ability to explore potential associations between these variables and the uptake of risk management measures and could have introduced bias into the analysis.

The retrospective, cross-sectional study design also presents challenges, particularly due to the absence of baseline PROs. It is difficult to assess participants’ body image, breast-related symptoms, or psychological well-being before opting for RRM or IBS. The single time-point assessment of quality of life, conducted at varying intervals following genetic counselling, complicates the interpretation of PROs, which are subject to fluctuations across time in this context.

The inclusion of participants based on their willingness to complete a questionnaire may have introduced volunteer bias, selecting carriers who do not represent the general population. Additionally, using a digital platform to administer the questionnaire might have selected a younger and more digitally apt population.

The guidelines for genetic testing and integration into the High-Risk Breast Clinic have changed over the patient’s follow-up time, so patients who could currently be part of the study population may have been missed. Additionally, cases where no active risk management strategy was chosen were not included in the analysis, particularly among individuals who did not engage with clinical care after a positive genetic test result.

The study's generalizability may be limited as it was conducted within a single institution, restricting its applicability to other populations with different contexts, even though our findings resemble those of other populations, as mentioned. A previous study demonstrated that physician specialty influences recommendations for decision-making, with surgical and medical oncologists more likely to recommend RRM compared to family practitioners and internists (Dhar et al. 2011). As the current cohort consisted of women advised by surgeons at a dedicated breast cancer centre, treatment bias could have been introduced.

## Conclusions

PROs were evaluated in carriers of P/LP variants in *BRCA1/2* genes who underwent either RRM or IBS. We observed that prior breast cancer history significantly influences decision-making. Although not significant, we noticed a slight trend toward higher *HADS* scores in the IBS group. The IBS group also scored higher in *BREAST-Q*. The key question is if the small reduction in depression and anxiety potentially associated with RRM justifies the possible small decline in quality of life, body image satisfaction, and sexual well-being for the individual carrier of a P/LP variant in *BRCA1/2* genes. As these differences were exclusively observed in the group with no personal history of breast cancer, we hypothesized these results may be due to the lack of perception of the true risk of developing breast cancer in carriers of P/LP variants in *BRCA1/2* genes. This highlights that shared decision-making and thorough informed consent discussions are crucial in ensuring carriers understand the benefits and drawbacks of RRM.

Future research should focus on obtaining baseline PROM scores and assessing the long-term effect and variation of different risk management strategies on quality-of-life and body image outcomes, to better assist carriers of P/LP variants in *BRCA1/2* genes regarding their cancer risk management.

## Data Availability

Data are not freely available as genetic data are sensitive data, for which we are obliged legally and ethically in our country to restrict the access due to preserving patient privacy.
